# Oral Transmissibility of Prion Disease Is Enhanced by Binding to Soil Particles

**DOI:** 10.1371/journal.ppat.0030093

**Published:** 2007-07-06

**Authors:** Christopher J Johnson, Joel A Pedersen, Rick J Chappell, Debbie McKenzie, Judd M Aiken

**Affiliations:** 1 Program in Cellular and Molecular Biology, University of Wisconsin-Madison, Madison, Wisconsin, United States of America; 2 Department of Comparative Biosciences, School of Veterinary Medicine, University of Wisconsin-Madison, Madison, Wisconsin, United States of America; 3 Department of Soil Science and Molecular and Environmental Toxicology Center, University of Wisconsin-Madison, Madison, Wisconsin, United States of America; 4 Biostatistics and Medical Informatics, University of Wisconsin Medical School, Madison, Wisconsin, United States of America; Roslin Institute, United Kingdom

## Abstract

Soil may serve as an environmental reservoir for prion infectivity and contribute to the horizontal transmission of prion diseases (transmissible spongiform encephalopathies [TSEs]) of sheep, deer, and elk. TSE infectivity can persist in soil for years, and we previously demonstrated that the disease-associated form of the prion protein binds to soil particles and prions adsorbed to the common soil mineral montmorillonite (Mte) retain infectivity following intracerebral inoculation. Here, we assess the oral infectivity of Mte- and soil-bound prions. We establish that prions bound to Mte are orally bioavailable, and that, unexpectedly, binding to Mte significantly enhances disease penetrance and reduces the incubation period relative to unbound agent. Cox proportional hazards modeling revealed that across the doses of TSE agent tested, Mte increased the effective infectious titer by a factor of 680 relative to unbound agent. Oral exposure to Mte-associated prions led to TSE development in experimental animals even at doses too low to produce clinical symptoms in the absence of the mineral. We tested the oral infectivity of prions bound to three whole soils differing in texture, mineralogy, and organic carbon content and found soil-bound prions to be orally infectious. Two of the three soils increased oral transmission of disease, and the infectivity of agent bound to the third organic carbon-rich soil was equivalent to that of unbound agent. Enhanced transmissibility of soil-bound prions may explain the environmental spread of some TSEs despite the presumably low levels shed into the environment. Association of prions with inorganic microparticles represents a novel means by which their oral transmission is enhanced relative to unbound agent.

## Author Summary

Transmissible spongiform encephalopathies (TSEs) are a group of incurable neurological diseases likely caused by a misfolded form of the prion protein. TSEs include scrapie in sheep, bovine spongiform encephalopathy (“mad cow” disease) in cattle, chronic wasting disease in deer and elk, and Creutzfeldt-Jakob disease in humans. Scrapie and chronic wasting disease are unique among TSEs because they can be transmitted between animals, and the disease agents appear to persist in environments previously inhabited by infected animals. Soil has been hypothesized to act as a reservoir of infectivity and to bind the infectious agent. In the current study, we orally dosed experimental animals with a common clay mineral, montmorillonite, or whole soils laden with infectious prions, and compared the transmissibility to unbound agent. We found that prions bound to montmorillonite and whole soils remained orally infectious, and, in most cases, increased the oral transmission of disease compared to the unbound agent. The results presented in this study suggest that soil may contribute to environmental spread of TSEs by increasing the transmissibility of small amounts of infectious agent in the environment.

## Introduction

Bovine spongiform encephalopathy, human Creutzfeldt-Jakob disease and kuru, sheep scrapie, and chronic wasting disease of deer, elk, and moose belong to the class of fatal, infectious neurodegenerative diseases known as transmissible spongiform encephalopathies (TSEs) or prion diseases [1,2]. The precise nature of the etiological agent of these diseases remains controversial, but most evidence points to a misfolded isoform of the prion protein (PrP^TSE^) as the major, if not sole, component of the pathogen [3].

Sheep scrapie and cervid (deer, elk, and moose) chronic wasting disease are distinct among TSEs because epizootics can be maintained by horizontal transmission from infected to naïve animals [4–6], and transmission is mediated, at least in part, by an environmental reservoir of infectivity [7–10]. The presence of an environmental TSE reservoir impacts several epidemiological factors including contact rate (the frequency animals come in contact with the disease agent), duration of exposure (time period over which animals come in contact with the pathogen), and the efficiency of transmission (the probability that an exposed individual contracts the disease).

The oral route of exposure appears responsible for environmental transmission of chronic wasting disease and scrapie [6,11]; the propagation of bovine spongiform encephalopathy epizootics (feeding TSE-infected meat and bonemeal to cattle); the appearance of variant Creutzfeldt-Jacob disease in humans and feline spongiform encephalopathy in cats (presumably by consumption of bovine spongiform encephalopathy–infected beef) [12,13]; the spread of kuru among the Fore of Papua New Guinea (ritualistic endocannibalism [14–16]); and outbreaks of transmissible mink encephalopathy (TME) in farm-reared mink [17]. Following consumption, TSE agent is sampled by gut-associated lymphoid tissue, such as Peyer's patches or isolated lymphoid follicles, and accumulates in lymphatic tissues before entering the central nervous system via the enteric nervous system [18–20]. While ingestion is a biologically relevant TSE exposure route, oral dosing is a factor of ~10^5^ less efficient than intracerebral inoculation in inducing disease in rodent models [21]. The amounts of TSE agent shed into the environment are presumably small. The assumed low levels of TSE agent in the environment and the inefficiency of oral transmission have led to uncertainty about the contribution of environmental reservoirs of infectivity to prion disease transmission.

We and others have hypothesized that soil may serve as a reservoir of TSE infectivity [8,9,22,23]. Deliberate and incidental ingestion of soil by ruminants can amount to hundreds of grams daily [24,25]. Prions enter soil environments via decomposition of infected carcasses [8,26], alimentary shedding [11,27,28], deliberate burial of diseased carcasses/material [29], and possibly, urinary excretion [30]. TSE agent persists for years when buried in soil [26]. The disease-associated prion protein sorbs to soil particles [22,31,32], and the interaction of PrP^TSE^ with the common aluminosilicate clay mineral montmorillonite (Mte) is remarkably avid [22]. Despite this strong binding, PrP^TSE^–Mte complexes are infectious when inoculated into brains of recipient animals [22].

For TSEs to be transmitted via ingestion of prion-contaminated soil, prions bound to soil components must remain infectious by the oral route of exposure. We therefore investigated the oral infectivity of Mte- and soil-bound prions. We examined the effects of prion source (viz. infected brain homogenate [BH] and purified PrP^TSE^) and dose on disease penetrance (proportion of animals eventually exhibiting clinical TSE symptoms) and incubation period (time to onset of clinical symptoms) in experiments with Mte. We investigated the oral infectivity of soil particle–bound prions to Syrian hamsters using four dosing regimes: (1) infected BH mixed with Mte (BH–Mte mixtures), (2) isolated complexes of purified PrP^TSE^ bound to Mte (PrP^TSE^–Mte complexes), (3) purified PrP^TSE^ mixed with Mte (PrP^TSE^–Mte mixtures), and (4) PrP^TSE^ mixed with each of three whole soils (PrP^TSE^–soil mixtures). The rationale for each dosing regime is described below. Survival analysis was used to assess risk of clinical disease manifestation and quantify differences in effective titer. Application of survival analysis to oral bioassays of TSE transmissibility is discussed in Figure S1 and Text S1.

## Results

### Oral Infectivity of BH–Mte Mixtures

**Figure 1:**
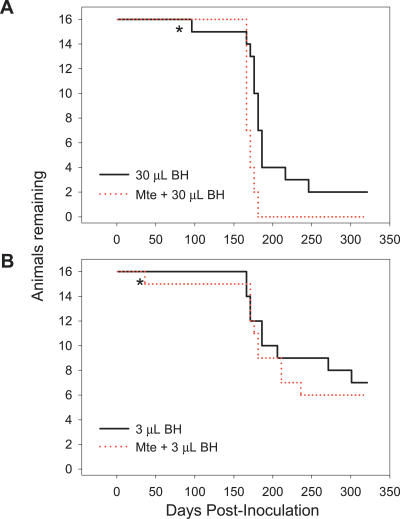
No Loss of Oral TSE Transmissibility Following Sorption of Prions from Infected BH to Mte (BH–Mte Mixtures) The oral transmissibility of prions in 30 (A) and 3 (B) μL was not diminished by dosing with Mte. * indicates non-TSE intercurrent death. Animals dosed with Mte alone remained healthy throughout the course of the experiment (unpublished data).

To examine the effect of Mte on the oral transmissibility of prions in BH, we incubated infected BH with clay particles for 2 h to allow sorption of the agent; controls lacking Mte were treated identically [22]. Three doses of 10% BH (30, 3, and 0.3 μL) were assayed. Diminished gastrointestinal bioavailability was expected to be evidenced by significant lengthening of incubation period, reduced disease penetrance, or both. Binding of either 30 or 3 μL of brain material to Mte yielded disease penetrance and incubation periods similar to BH alone (Figure 1A and 1B), a finding consistent with our previous report that a substantial fraction of PrP^TSE^ in clarified BH binds to Mte and that Mte-bound prions remain infectious [22].

**Figure 2:**
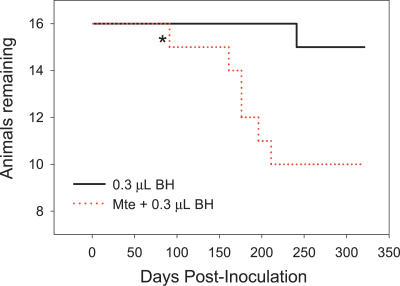
Mte Enhances Oral TSE Transmission at a Low Dose of Infected BH (BH–Mte Mixtures) Ingestion of Mte mixed with a lower dose of TSE-infected BH (0.3 μL) markedly shortens incubation period and increases disease penetrance relative to an equal amount of unbound BH. * indicates non-TSE intercurrent death. Animals dosed with Mte alone remained healthy throughout the course of the experiment (unpublished data).

Surprisingly, at the lowest BH dose (0.3 μL, Figure 2), sorption of TSE agent to Mte enhanced transmission, increasing disease penetrance and shortening incubation period. Adjusted for the amount of BH administered and combined across doses, Mte significantly enhanced oral transmissibility (*p* < 0.0001). Survival analysis indicated the risk of clinical disease manifestation relative to Mte-free controls was 3.03 (95% confidence interval [CI]: 1.68, 5.45), signifying an increase in the effective titer of TSE agent. While the influence of Mte was significant when tested across all BH doses, the effect was most readily observed at 0.3 μL. The dose-dependent difference in the influence of Mte on transmissibility may be attributable to competition between macromolecules in BH (e.g., lipids, other proteins, nucleic acids) with PrP^TSE^ for sorption sites on the clay surface. Such competition was evidenced by detection of unbound PrP^TSE^ and other proteins in incubations of Mte with 30 and 3 μL BH (unpublished data).

### Oral Infectivity of PrP^TSE^–Mte Complexes

To examine the influence of Mte on oral transmissibility without the interference of other macromolecules from brain homogenate, we purified PrP^TSE^ and inoculated hamsters using two different dosing regimes. The first dosing regime (PrP^TSE^–Mte complexes) was designed to directly assay the infectivity of PrP^TSE^ sorbed to Mte surfaces (i.e., the amount of unbound PrP^TSE^ was minimized in treatments containing Mte). Purified PrP^TSE^ was clarified to remove large aggregates, and after 2-h incubation with Mte, PrP^TSE^–Mte complexes were separated from unbound protein by centrifugation through a sucrose cushion [22]. Hamsters were orally challenged with the isolated PrP^TSE^–Mte complexes [22] or an amount of unbound clarified PrP^TSE^ (200 or 20 ng) equivalent to that introduced into the clay suspension (Table 1). Immunoblot analysis of the inocula (Figure S2A) demonstrated that the amount of PrP in the unbound samples was not less than that in PrP^TSE^–Mte complexes.

**Table 1:**
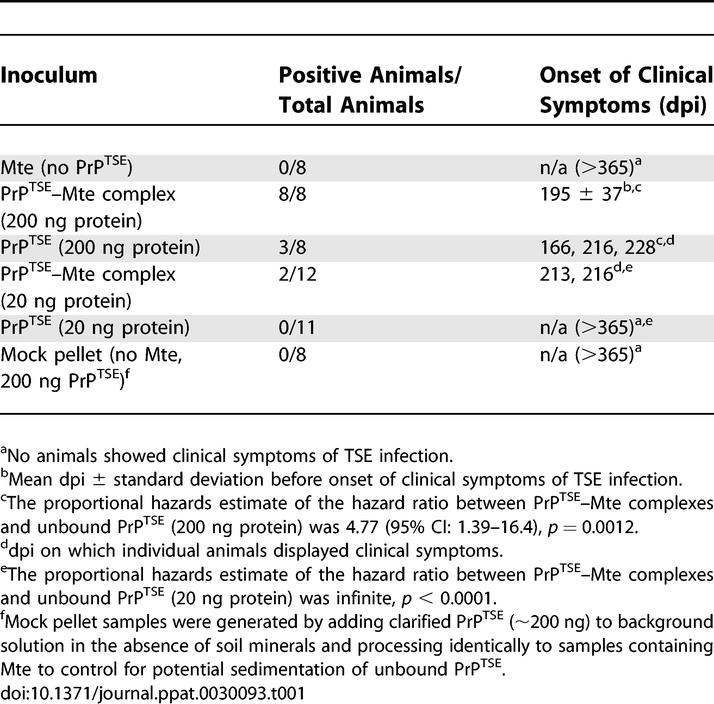
Prions Adsorbed to Mte Clay Are Infectious Perorally

Sorption of PrP^TSE^ to Mte dramatically enhanced prion disease transmission (Table 1). Approximately 38% of animals receiving 200 ng of unbound clarified PrP^TSE^ exhibited clinical symptoms with an incubation period for infected animals of 203 ± 33 (mean ± standard deviation) days post inoculation (dpi). In contrast, all animals orally dosed with an equivalent amount of Mte-bound PrP^TSE^ manifested disease symptoms (incubation period = 195 ± 37 dpi), an enhancement of transmission comparable to that observed for the lowest BH dose (Figure 2). Animals inoculated with Mte alone or 10-fold less unbound clarified PrP^TSE^ (20 ng) remained asymptomatic throughout the course of the experiment (>365 dpi), whereas 20 ng of clarified PrP^TSE^ adsorbed to Mte produced TSE infection in 17% of animals. These data establish not only that the Mte-bound prions remain infectious via the oral route of exposure, but that agent binding to Mte increases disease penetrance, enhancing the efficiency of oral transmission.

### Oral Infectivity of PrP^TSE^–Mte Mixtures

The second oral dosing regime using purified PrP^TSE^ (PrP^TSE^–Mte mixtures) was designed to ensure that treatments with and without Mte contained equivalent PrP^TSE^ doses. These experiments differed from those above in two important aspects. First, PrP^TSE^–Mte complexes were not separated from suspension prior to inoculation so that comparable amounts of infectious agent were administered to both treatment groups. In the first dosing regime, some PrP^TSE^ may have been lost during sedimentation of PrP^TSE^–Mte complexes (Figure S2A). Second, the purified prion preparation was not clarified and therefore contained a range of PrP^TSE^ aggregate sizes. The sizes of PrP^TSE^ aggregates attached to Mte particles were expected to be more heterogeneous than those in the first dosing regime.

**Figure 3:**
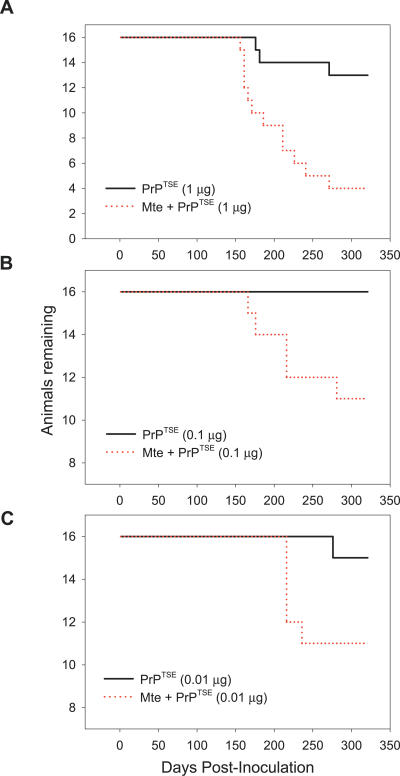
Concurrent Peroral Administration of Mte and PrP^TSE^ Dramatically Increases Disease Penetrance at Agent Doses That Typically Fail to Produce Clinical Symptoms (PrP^TSE^–Mte Mixture) (A) Mte increases disease penetrance and shortens incubation periods associated with ingestion of 1 μg of purified PrP^TSE^. Concurrent peroral dosage of lower, typically subclinical doses of purified PrP^TSE^ (0.1 or 0.01 μg, [B and C]) with Mte increases disease incidence. Animals dosed with Mte alone remained healthy throughout the course of the experiment (unpublished data).

Compared to Mte-free controls, administration of purified PrP^TSE^ mixed with Mte increased disease penetrance at all doses and shorted incubation times in the 1-μg PrP^TSE^ treatment (Figure 3A). At the two lower doses (0.1 and 0.01 μg PrP^TSE^), binding of the agent to Mte dramatically increased disease penetrance (31%) at PrP^TSE^ doses failing to yield clinical infection in 31 of 32 animals in the absence of the clay mineral (Figure 3B and 3C). Comparison of the survival curves in Figure 3A and 3C indicates that the 0.01-μg PrP^TSE^–Mte mixture was at least as infectious as 1-μg PrP^TSE^ Mte-free samples, suggesting that sorption of purified PrP^TSE^ to Mte enhanced transmission by a factor of ≥100.

To quantify the contributions to changes in relative risk of prion dose and agent sorption to Mte, we constructed a multivariate Cox proportional hazards model with two covariates: log_10_ PrP^TSE^ dose and Mte presence (Table 2). Each log_10_ increase in PrP^TSE^ dose multiplies the relative risk by a factor of ~2 (i.e., a 10-fold increase in dose approximately doubles the risk of infection). Notably, sorption of purified PrP^TSE^ to Mte multiplies the relative risk by a factor of ~8. These values allowed computation of a multiplicative equivalence factor between PrP^TSE^ dose and Mte presence in the inoculum. Expressed in terms of PrP^TSE^ dose, addition of Mte to the inoculum is equivalent to multiplying the PrP^TSE^ dose by a factor of 680 (95% CI 16, ∞); that is, inclusion of Mte increases the effective titer of a given PrP^TSE^ dose by 680-fold. Estimates of effective titer span a wide range (95% CI 16, ∞), and the present data do not allow us to place an upper bound on the increased risk associated with the presence of Mte in a sample. At a minimum, effective titer increased by 1.2 orders of magnitude, but the effect could be substantially larger. The best estimate of the Cox analysis represents a 2.8 order-of-magnitude increase in effective titer.

**Table 2:**
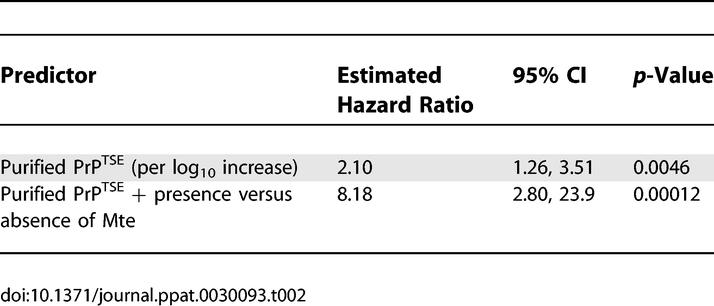
Estimated Hazard Ratios due to Prion Dose and Mte Addition

### Strain Properties

Oral administration of Mte-bound PrP^TSE^ did not appear to alter strain properties. Following limited proteinase K (PK) digestion, many PrP^TSE^ strains can be discriminated by the size and glycoform pattern of PK-resistant core of PrP^TSE^ (PrP-res) [33–36]. Strain differences are also manifested in specific clinical symptoms. At the conclusion of the oral transmission experiments described above, the brains of clinically infected animals were assayed for PrP-res by immunoblotting (Figure S3). Differences in the molecular mass and glycoform distribution of PrP-res were not apparent between the treatment groups. Furthermore, clinical presentation of disease (symptoms or length of clinically positive period) did not differ between treatments.

**Figure 4:**
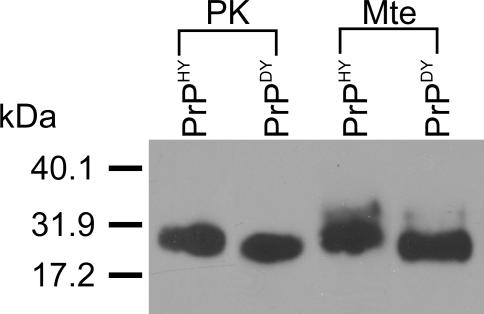
Maintenance of Strain Properties for Mte-Associated PrP^TSE^ BH from hamsters clinically affected with either HY or DY agents were incubated with Mte to allow binding. Desorbed proteins were analyzed by SDS-PAGE and immunoblotting. Cleavage patterns of PrP^HY^ and PrP^DY^ extracted from Mte parallel PK cleavage patterns for the respective proteins: cleaved PrP^DY^ migrates further (corresponding to a 1- to 2-kDa molecular mass difference) than cleaved PrP^HY^. Immunoblot used the PrP-specific antibody 3F4.

The experiments described above were conducted using the Hyper (HY) strain of hamster-adapted TME agent (PrP^HY^). To further examine the strain stability of Mte-bound PrP^TSE^, we employed the Drowsy (DY) strain of hamster-passaged TME agent (PrP^DY^) to investigate the molecular mass of PrP desorbed from Mte and the effect of this clay mineral on oral transmissibility [35,36]. We previously reported the N-terminal cleavage of PrP^HY^ extracted from Mte yielding a product similar in size to PK-digested PrP^HY^ [22]. PK digestion of PrP^HY^ and PrP^DY^ results in products of characteristically different molecular masses [35,36]: the length of the PrP^HY^ digestion product exceeds that of PrP^DY^ by at least ten amino acids [35,36]. We found that extraction of bound PrP^DY^ from Mte resulted in a product similar in molecular mass to PrP^DY^ cleaved by PK (Figure 4). These data are consistent with the idea that strain properties are preserved when PrP^TSE^ binds to Mte. DY agent is not orally transmissible [37], and we find that sorption of DY to Mte does not facilitate oral transmission (Text S1).

### Oral Transmission of PrP^TSE^ Bound to Whole Soils

**Figure 5:**
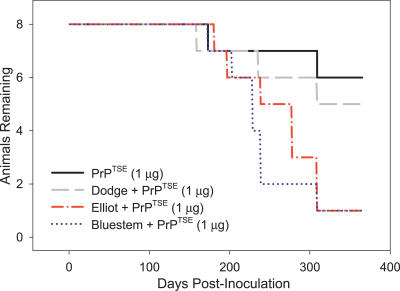
Prions Bound to Whole Soils Remain Orally Infectious and Some Soils Increase Transmission Three soils (Dodge, Elliot, and Bluestem) were incubated in the presence of purified PrP^TSE^. The samples were orally dosed into hamsters and found to remain orally infectious. Agent association with Elliot and Bluestem soils increases disease incidence, whereas Dodge soil does not influence disease transmission. Animals dosed with soil alone remained healthy throughout the course of the experiment (unpublished data).

Natural soils are composed of a complex mixture of inorganic and organic components of various particle sizes. Smectitic clays such as Mte are important constituents of many natural soils and contribute significantly to their surface reactivity [38]. In natural soils, metal oxide and organic matter often coat smectite surfaces and may alter their propensity to bind PrP^TSE^. Furthermore, additional sorbent phases may be important in the binding of TSE agents to whole soils. We previously demonstrated that PrP^TSE^ binds to whole soils of varying texture, mineralogy, and organic carbon content [22]. To examine the impact of agent binding to whole soil on oral TSE transmission, we incubated 1 μg of purified PrP^TSE^ with each of three whole soil samples (Elliot, Dodge, and Bluestem soils) to allow sorption, and then orally dosed hamsters with the PrP^TSE^–soil mixtures. Soil-bound TSE agent remained infectious perorally, and two of the soils significantly enhanced oral disease transmission (Figure 5). Hazard ratios between Elliot (4.76 [95% CI: 1.38–16.4], *p* = 0.019) and Bluestem (6.04 [95% CI: 1.59–22.9], *p* = 0.013) soils and unbound PrP^TSE^ indicate a significant increase in transmissibility, but no difference for the Dodge soil (1.66 [95% CI: 0.52–1.66], *p* = 0.578). The hazard ratios for the Elliot and Bluestem soils did not differ from one another (0.79 [95% CI: 0.19–3.25], *p* = 0.543) indicating statistical equivalence in transmissibility. The limited numbers of animals in the treatment groups precluded derivation of a multiplicative equivalence factor to equate the presence of Elliot or Bluestem soil with dose of infectious agent; however, substantially more animals in the Elliot and Bluestem treatment groups (14 of 16 animals, 87.5% penetrance) displayed clinical symptoms compared to the unbound PrP^TSE^ treatment group (two of eight animals, 25% penetrance).

## Discussion

These experiments address the critical question of whether soil particle–bound prions are infectious by an environmentally relevant exposure route, namely, oral ingestion. Oral infectivity of soil particle–bound prions is a *conditio sine qua non* for soil to serve as an environmental reservoir for TSE agent. The maintenance of infectivity and enhanced transmissibility when TSE agent is bound to the common soil mineral Mte is remarkable given the avidity of the PrP^TSE^–Mte interaction [22]. One might expect the avid interaction of PrP^TSE^ with Mte to result in the mineral serving as a sink, rather than a reservoir, for TSE infectivity. Our results demonstrate this may not be the case. Furthermore, sorption of prions to complex whole soils did not diminish bioavailability, and in two of three cases promoted disease transmission by the oral route of exposure. While extrapolation of these results to environmental conditions must be made with care, prion sorption to soil particles clearly has the potential to increase disease transmission via the oral route and contribute to the maintenance of TSE epizootics.

Two of three tested soils potentiated oral prion disease transmission. The reason for increased oral transmissibility associated with some, but not all, of the soils remains to be elucidated. One possibility is that components responsible for enhancing oral transmissibility were present at higher levels in the Elliot and Bluestem soils than in the Dodge soil. The major difference between the Dodge soil and the other two soils was the extremely high natural organic matter content of the former (34%, [22]). The Dodge and Elliot soils contained similar levels of mixed-layer illite/smectite, although the contribution of smectite layers was higher in the Dodge soil (14%–16%, [22]). The organic matter present in the Dodge soil may have obstructed access of PrP^TSE^ to sorption sites on smectite (or other mineral) surfaces.

The mechanism by which Mte or other soil components enhances the oral transmissibility of particle-bound prions remains to be clarified. Aluminosilicate minerals such as Mte do not provoke inflammation of the intestinal lining [39]. Although such an effect is conceivable for whole soils, soil ingestion is common in ruminants and other mammals [25]. Prion binding to Mte or other soil components may partially protect PrP^TSE^ from denaturation or proteolysis in the digestive tract [22,40] allowing more disease agent to be taken up from the gut than would otherwise be the case. Adsorption of PrP^TSE^ to soil or soil minerals may alter the aggregation state of the protein, shifting the size distribution toward more infectious prion protein particles, thereby increasing the specific titer (i.e., infectious units per mass of protein) [41]. In the intestine, PrP^TSE^ complexed with soil particles may be more readily sampled, endocytosed (e.g., at Peyer's patches), or persorbed than unbound prions. Aluminosilicate (as well as titanium dioxide, starch, and silica) microparticles, similar in size to the Mte used in our experiments, readily undergo endocytotic and persorptive uptake in the small intestine [42–44]. Enhanced translocation of the infectious agent from the gut lumen into the body may be responsible for the observed increase in transmission efficiency.

Survival analysis indicated that when bound to Mte, prions from both BH and purified PrP^TSE^ preparations were more orally infectious than unbound agent. Mte addition influenced the effective titer of infected BH to a lesser extent than purified PrP^TSE^. Several nonmutually exclusive factors may explain this result: (1) other macromolecules present in BH (e.g., lipids, nucleic acids, other proteins) compete with PrP^TSE^ for Mte binding sites; (2) prion protein is more aggregated in the purified PrP^TSE^ preparation than in BH [45], and sorption to Mte reduces PrP^TSE^ aggregate size, increasing specific titer [41]; and (3) sorption of macromolecules present in BH to Mte influences mineral particle uptake in the gut by altering surface charge or size, whereas the approximately 1,000-fold lower total protein concentration in purified PrP^TSE^ preparations did not produce this effect.

We previously showed that other inorganic microparticles (kaolinite and silicon dioxide) also bind PrP^TSE^ [22]. All three types of microparticles are widely used food additives and are typically listed as bentonite (Mte), kaolin (kaolinite), and silica (silicon dioxide). Microparticles are increasingly included in Western diets. Dietary microparticles are typically inert and considered safe for consumption by themselves, do not cause inflammatory responses or other pathologies, even with chronic consumption, and are often sampled in the gut and transferred from the intestinal lumen to lymphoid tissue [39,46,47]. Our data suggest that the binding of PrP^TSE^ to dietary microparticles has the potential to enhance oral prion disease transmission and warrants further investigation.

In conclusion, our results provide compelling support for the hypothesis that soil serves as a biologically relevant reservoir of TSE infectivity. Our data are intriguing in light of reports that naïve animals can contract TSEs following exposure to presumably low doses of agent in the environment [5,7–9]. We find that Mte enhances the likelihood of TSE manifestation in cases that would otherwise remain subclinical (Figure 3B and 3C), and that prions bound to soil are orally infectious (Figure 5). Our results demonstrate that adsorption of TSE agent to inorganic microparticles and certain soils alter transmission efficiency via the oral route of exposure.

## Materials and Methods

### TSE agent source.

Syrian hamsters (cared for according to all institutional protocols) were experimentally infected with the HY or DY strain of hamster-adapted TME agent [48]. Brain homogenate, 10% w/v, was prepared in 10 mM NaCl. PrP^TSE^ was purified to a P_4_ pellet from brains of hamsters infected with the HY strain using a modification of the procedure described by Bolton et al. [49,50]. The P_4_ pellet prepared from four brains was resuspended in 1 mL of 10 mM Tris (pH 7.4) with 130 mM NaCl. In the subset of experiments using PrP^TSE^–Mte complexes, larger prion aggregates were removed from the preparation by collecting supernatants from two sequential 5-min centrifugations at 800 *g* (clarification). Protein concentrations were determined using the Bio-Rad (http://www.bio-rad.com) DC protein assay as directed by the manufacturer's instructions.

### Preparation of inocula and oral dosing.

Four types of Mte- or soil-containing inocula were prepared: BH–Mte mixtures, PrP^TSE^–Mte mixtures, PrP^TSE^–soil mixtures, and PrP^TSE^–Mte complexes (see below). To prepare mixtures of BH or PrP^TSE^ with Mte, the indicated amount of 10% brain homogenate (Figures 1 and 2) or PrP^TSE^ (Figure 3) was added to 500 μL of 10 mM NaCl in the presence or absence of 500 μg of Na^+^-saturated Mte (particle hydrodynamic diameter = 0.5–2 μm) (prepared per [51]). Mixtures of PrP^TSE^ and whole soils (Figure 5) were prepared by adding 1 μg of PrP^TSE^ to 500 μL of 5 mM CaCl_2_ in the presence or absence of 1 mg of each soil type. Samples were rotated at ambient temperature for 2 h, like samples were pooled, and the equivalent of 500 μg of Mte or 1 mg of whole soil was orally inoculated into each hamster. We previously showed that absorption of purified PrP^TSE^ to Mte was complete within 2 h [22].

Isolated PrP^TSE^–Mte complexes were prepared as previously described [22]. Briefly, the indicated amount of clarified PrP^TSE^ (200 or 20 ng, Table 1) was added to 500 μg of Mte in 10 mM NaCl (500 μL final volume) per sample. Mixtures were rotated at ambient temperature for 2 h. Each PrP^TSE^–Mte suspension was placed over a 750-mM sucrose cushion prepared in 10 mM NaCl and centrifuged at 800 *g* for 7 min to sediment mineral particles and adsorbed PrP^TSE^. PrP^TSE^–Mte complexes were resuspended in 500 μL of 10 mM NaCl and pooled. The equivalent of 500 μg of Mte was orally inoculated into each hamster. To control for potential sedimentation of unbound PrP^TSE^, “mock” samples lacking Mte were processed identically, and any sedimented material was inoculated into hamsters. As a positive control, unbound PrP^TSE^ (200 or 20 ng) was orally administered to hamsters. All oral inoculations were via pipette and voluntary consumption. Following oral dosing, hamsters were observed twice weekly for the onset of clinical symptoms [48] for at least 300 d, a period of time found sufficient to observe most or all clinical cases [52].

### Immunoblotting.

Immunoblotting was performed as previously described [22]. Briefly, proteins were separated by SDS-PAGE (4%–20% gradient for analysis of inocula, 15% for analysis of brain PrP), transferred to polyvinyl difluoride membranes, and immunoblotted with the PrP-specific antibody 3F4 (1:40,000 dilution). Detection was achieved with HRP-conjugated goat anti-mouse immunoglobulin G.

### Analysis of PrP^TSE^ inocula.

The quantity and characteristics of PrP^TSE^ dosed in Table 1 and Figure 3 were compared by immunoblot analysis (Figure S2A and S2B). For both unbound and Mte-bound PrP^TSE^ inocula, a 50- μL aliquot (one-tenth the total volume) of each 200-ng or 1-μg sample of PrP^TSE^ (Figure 3 and Table 1, respectively) was removed following the 2-h incubation. Samples with 20 ng PrP^TSE^ were not consistently detectable by immunoblot analysis. Mte was sedimented by 1-min centrifugation at 14,000 *g,* and PrP was extracted for 10 min in 5 μL of 10× sample buffer (100 mM Tris [pH 8.0], 10% SDS, 7.5 mM EDTA, 100 mM dithiothreitol, and 30% glycerol) at 100 °C. While still hot, Mte was sedimented by brief centrifugation, and the supernatant containing extracted PrP was diluted with 10 mM NaCl to a total volume of 50 μL. Sample buffer was added to the unbound PrP^TSE^ samples to a 1× final concentration, and samples were heated at 100 °C for 10 min prior to SDS-PAGE and immunoblotting. Analysis of the sorption of PrP^HY^ and PrP^DY^ from brain homogenate to Mte was performed as previously described [22].

### Analysis of PK-resistant PrP.

Brains from hamsters orally dosed with unbound PrP^HY^ or Mte-bound PrP^HY^ were homogenized to 10% w/v in PBS. For samples without PK, 10 μL of BH was mixed 1:1 with 10× sample buffer and heated at 100 °C for 5 min. Other samples (30 μL) were treated with PK (50 μg·mL^−1^) for 30 min at 37 °C. Phenylmethylsulfonyl fluoride was added to achieve a concentration of 1 mM to block PK activity, and samples were diluted 1:1 with 10× sample buffer and heated at 100 °C for 5 min prior to SDS-PAGE and immunoblotting.

### Survival analysis.

Multivariate Cox proportional hazards regressions [53] were used to estimate the effects of PrP^TSE^ dose (using log_10_ PrP^TSE^ dose as a continuous variable) and Mte inclusion on times to onset of clinical symptoms [54]. Several diagnostic procedures were performed to assess the validity of the Cox regressions. First, interaction in the statistical model between Mte and PrP^TSE^ dose was tested and found to be far from significant (*p* = 0.92); this interaction was therefore excluded from further consideration. Second, comparison of the linear fit with the three-level dose factor indicated that the nonlinearity of the log_10_ prion dose covariate was nonsignificant (*p* = 0.21); log_10_ prion dose was therefore retained as a continuous covariate. Last, cumulative hazard curves were approximately parallel and simple diagnostics for proportionality [53] showed the assumption of linearity to be appropriate.

Equivalence factors (the dose multiplier equal in effect to adding Mte to a sample) can be derived as 10 raised to the inverse ratio of the Mte and dilution coefficients. A 95% CI for the ratio was generated using Fieller's method and then exponentiated to produce a CI for the factor [53]. All Cox analyses were performed using S-PLUS version 3.4 [55].

## Supporting Information

Figure S1 Typical Survival Curves for Intracerebral and Oral Inoculation of a Dilution Series of Hamster-Adapted Scrapie Agent (Prion Strain 263K) in 10% BH For the intracerebral inoculation (inset), each log_10_ dilution leads to a characteristic lengthening in incubation period (adapted from Prusiner et al. [56]). Lines represent the mean incubation period of eight hamsters. Typical survival curves following peroral administration of 263K prions over a partially overlapping range of doses reveal that each log_10_ dilution lengthens the incubation period and reduces disease penetrance (adapted from Baier et al. [52]).
(22 KB PDF)

Figure S2 Immunoblot Analysis of Inocula for Experiments Examining PrP^TSE^−Mte Complexes and PrP^TSE^−Mte Mixtures Aliquots of PrP^TSE^−Mte complexes and corresponding clarified PrP^TSE^ inocula were analyzed by immunoblotting to assess whether processing losses could account for the reduced transmission by unbound PrP^TSE^ compared to the Mte-bound agent. One-tenth (50 μL) of the (A) 200 ng clarified PrP^TSE^ ± Mte samples or (B) 1 μg PrP^TSE^ + Mte and corresponding unbound PrP^TSE^ samples were analyzed by immunoblotting. In both cases the Mte-bound samples contained less or similar amounts of protein compared to the unbound samples.
(25.7 MB TIF)

Figure S3 PK-Resistant PrP from the Brains of Clinically Infected Animals Dosed with Unbound PrP^TSE^ and PrP^TSE^−Mte Mixtures At the conclusion of the transmission experiments, brain homogenates from clinically diseased hamsters were analyzed by SDS-PAGE and immunoblotting with the PrP-specific antibody 3F4. Brains from animals dosed with unbound PrP^TSE^ and PrP^TSE^−Mte mixtures were assayed. Similar PrP molecular masses and banding patterns of both uncleaved and PK-treated PrP were observed in both treatments.
(17 MB TIF)

Text S1 Survival Analysis in Oral TSE Infection, and Oral Administraion of Drowsy Agent with Mte (37 KB DOC)

### Accession Number

The GenBank (http://www.ncbi.nlm.nih.gov) accession number for PrP is M14054.

## Abbreviations

BH: brain homogenate
CI: confidence interval
dpi: day post-inoculation
Mte: montmorillonite
PK: proteinase K
PrP: prion protein
PrP^DY^: Drowsy TSE agent–associated prion protein
PrP^HY^: Hyper TSE agent–associated prion protein
PrP-res: proteinase K–resistant PrP
PrP^TSE^: disease-associated prion protein
TME: transmissible mink encephalopathy
TSE: transmissible spongiform encephalopathy
