# Supplementary material for: Oral Transmissibility of Prion Disease Is Enhanced by Binding to Soil Particles
Source: PLoS Pathog. 2007 Jul 6;3(7):e93. doi: 10.1371/journal.ppat.0030093 (PMC1904474; doi:10.1371/journal.ppat.0030093)
Supplement: Supplementary file 1 [file ppat.0030093.sg001.pdf]

Figure S1

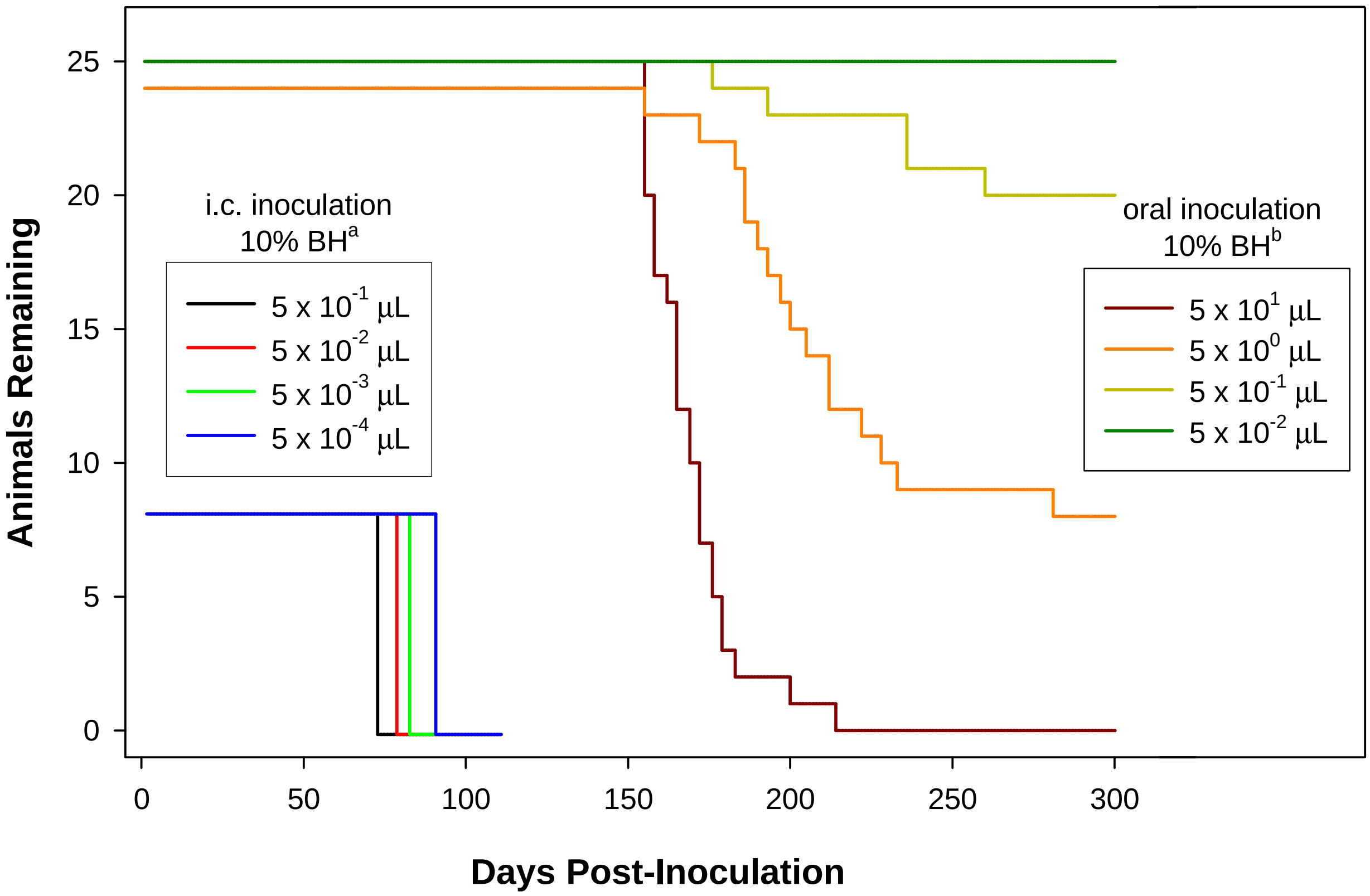
Animals Remaining
25
20
15
10
5
0
0
50
100
150
200
250
300
Days Post-Inoculation
i.c. inoculation
10% BH[a]
5 x 10^-1 µL
5 x 10^-2 µL
5 x 10^-3 µL
5 x 10^-4 µL
oral inoculation
10% BH[b]
5 x 10^1 µL
5 x 10^0 µL
5 x 10^-1 µL
5 x 10^-2 µL
