# Supplementary material for: Oral Transmissibility of Prion Disease Is Enhanced by Binding to Soil Particles
Source: PLoS Pathog. 2007 Jul 6;3(7):e93. doi: 10.1371/journal.ppat.0030093 (PMC1904474; doi:10.1371/journal.ppat.0030093)
Supplement: Supplementary file 2 [file ppat.0030093.sd001.doc]

**Text S1: Survival Analysis in Oral TSE Infection**

Survival analysis is used to evaluate time-to-event data [1] and compare the relative risk/benefit of treatments based on the number of respondents and the time to response. While this type of analysis is exhaustively reviewed elsewhere [1], we briefly outline the use of survival analysis in the evaluation of animal bioassay data. We preface our description of the non-parametric statistical analysis performed with a comparison of two typical routes of inoculation used in TSE studies to illustrate the need for survival analysis to interpret oral bioassay data.

Two frequently used TSE agent inoculation routes in laboratory animals are intracerebral (i.c.) injection and peroral dosing. In i.c. inoculations, the brain is directly exposed to infectious agent, and animals manifest clinical symptoms following a lengthy incubation period. The time to onset of clinical symptoms (number of days post inoculation (dpi)) relates directly to the infectious titer of the inoculum [2-4]. Infectious titer can be calculated in a relatively straightforward manner by this approach (incubation time interval assay). Typical i.c. inoculation data for a dilution series of infectious agent (adapted from Prusiner et al. [3] are presented in Figure S1 (inset). Each log_10_ reduction in agent titer produces a characteristic lengthening in incubation period. Until the dose approaches 1 ID_50, i.c._ (i.e., the dose causing disease in half the exposure group via i.c. inoculation), all animals eventually manifest clinical symptoms despite the lengthening of incubation period. Risk of disease manifestation relates to titer in a straightforward manner, and titer can be approximated by the linear formulas described elsewhere [2]. Non-parametric analyses of i.c. bioassays are often unnecessary and rarely employed (for an exception to this, see Peretz et al., 2006 [5]).

In laboratory rodent models, TSE transmission via oral route of exposure is much less efficient that i.c. inoculation (by a factor of ~10^5^ [6]) complicating interpretation of bioassay data. Oral inoculation is more biologically relevant than i.c. injection because ingestion of prion-contaminated materials is an important exposure route for most TSEs [7]. Difficulties in interpretation arise because inefficient disease transmission manifests itself in larger variance in the time to onset of clinical symptoms and, at low doses, incubation periods exceeding the lifetimes of experimental animals, reducing disease penetrance. Furthermore, the dynamic range of i.c. inoculation extends at least 4 orders of magnitude [2], whereas that of oral bioassay is substantially narrower. These factors conspire to complicate determination of starting titer from oral bioassays. Typical oral inoculation data for a dilution series of infectious agent (adapted from Baier et al. [8]) is presented in Figure S1. The highest dose shown (50 μL) results in an incubation period of 169 ± 14 dpi. Increasing dose beyond 50 μL fails to further shorten the incubation period. The oral ID_50_ is approached as the oral dose is decreased by only one order of magnitude. Further decreases in dose lengthen time-to-symptoms and reduce disease penetrance (Figure S1). Because risk of infection is asymmetrically distributed, description of oral bioassay survival curves with parametric statistics is not appropriate. Furthermore, non-TSE intercurrent deaths can complicate analysis of lengthy experiments such as those measuring oral transmissibility, and for low doses of agent, the lifespans of laboratory animals limit the duration (and hence, sensitivity) of the assays.

Survival analysis estimates the relative risk of disease acquisition based on the time to clinical manifestation and the number of animals exhibiting symptoms. In the present study, changes in relative risk were depicted with Kaplan-Meier curves and analyzed using log-rank tests. Proportional hazards models were used to adjust for dose and to estimate relative risks for the effects of dose and Mte addition. Change in relative risk is not a direct measure of titer, but rather a comparison of titers among samples (i.e., absolute titers are unknown). This type of analysis is most appropriate for treatment groups where the determination of the median (or some other descriptive quantile) is not possible.

As noted in the main text, survival analysis utilizes all available information in the study, including data from censored events (i.e., animals not exhibiting clinical symptoms). One consequence is that means and statistical procedures using or extending them such as *t*-tests, regressions, and analyses of variance, are not computable. All these tests require complete information on each subject. The most common alternative, used here, is to compare survival curves at each point in time using the ratios of their standardized slopes. When this ratio can be assumed constant over time (“proportional hazards”), it is referred to as the relative risk of or hazard ratio for infection. The Cox proportional hazards model estimates the relative risk and, as here, can estimate both at once, each variable adjusted for the other. Our model, described and justified in the manuscript, assumes a log-linear increase in hazard with dose and simultaneously fits the effect of Mte adjusted for dose. As described in the main text, the assumptions of proportional hazards and log-linear increase in hazard with dose appear to be met by our data set.

Although hazard ratios have direct relevance in some situations (e.g., when a study is conducted on a population of interest and performed in a realistic setting), the laboratory conditions in our experiments are clearly artificial. Additionally, experimental animals were chosen for reasons of practicality rather than interest. Therefore, we rendered our Mte results as relevant as possible by framing them in terms of a dose-equivalence factor. Thus, we can say not only that the estimated hazard ratio due to prion dose is 2.1 per log_10_ increase and that due to Mte addition is 8.2 in these animals under the experimental conditions, but we can use these estimates to answer the question "What increase in dose would be needed to boost transmission as much as Mte appears to do?" The estimated answer is large (though admittedly variable, with a wide confidence interval, the upper bound of which we are unable to define with the data from our study): adding Mte was equivalent to multiplying the TSE agent titer by a factor of 680. The application of this figure to other species raised under more natural conditions remains to be investigated. Our intent is to present the most applicable possible formulation of our results given the necessary constraints of the study.

**Oral Adminstration of Drowsy TSE agent with Mte**

We orally inoculated unbound (*n* = 8) and Mte-bound (*n* = 24) Drowsy agent (30 *μ*L 10% BH) into hamsters. At >475 dpi, none of these animals have presented with clinical signs of disease suggesting that agent sorption to Mte does not alter DY strain properties as to overcome the barrier to oral transmission. At the high DY brain homogenate dose tested, competition with other macromolecules for sorption sites on the clay mineral surface may have obscured the effect of Mte. When bound to Mte, purified PrP^HY^ increased oral disease transmission more that infected BH (Figures 1 and 3). Our Cox model, however, indicated that across all doses of BH, Mte increased the oral transmissibility of disease suggesting that an effect could be noted.

**References**

1.	Cox DR, Oakes D (1984) Analysis of survival data. London ; New York: Chapman and Hall. 201 p.

2.	Prusiner SB, Groth DF, Cochran SP, Masiarz FR, McKinley MP, et al. (1980) Molecular properties, partial purification, and assay by incubation period measurements of the hamster scrapie agent. Biochemistry 19: 4883-4891.

3.	Prusiner SB, Cochran SP, Downey DE, Groth DF (1981) Determination of scrapie agent titer from incubation period measurements in hamsters. Adv Exp Med Biol 134: 385-399.

4.	Prusiner SB, Cochran SP, Groth DF, Downey DE, Bowman KA, et al. (1982) Measurement of the scrapie agent using an incubation time interval assay. Ann Neurol 11: 353-358.

5.	Peretz D, Supattapone S, Giles K, Vergara J, Freyman Y, et al. (2006) Inactivation of prions by acidic sodium dodecyl sulfate. J Virol 80: 322-331.

6.	Kimberlin RH, Walker CA (1989) Pathogenesis of scrapie in mice after intragastric infection. Virus Res 12: 213-220.

7.	Shmakov AN, Ghosh S (2001) Prion proteins and the gut: Une liaison dangereuse? Gut 48: 443-447.

8.	Baier M, Norley S, Schultz J, Burwinkel M, Schwarz A, et al. (2003) Prion diseases: Infectious and lethal doses following oral challenge. J Gen Virol 84: 1927-1929.
